# Numerical and Experimental UAV Structure Investigation by Pre-Flight Load Test

**DOI:** 10.3390/s20113014

**Published:** 2020-05-26

**Authors:** Artur Kurnyta, Wojciech Zielinski, Piotr Reymer, Krzysztof Dragan, Michal Dziendzikowski

**Affiliations:** Air Force Institute of Technology, Airworthiness Division, 01-494 Warsaw, Poland; wojciech.zielinski@itwl.pl (W.Z.); piotr.reymer@itwl.pl (P.R.); krzysztof.dragan@itwl.pl (K.D.); michal.dziendzikowski@itwl.pl (M.D.)

**Keywords:** UAV, load test, structural integrity, numerical simulation, panel method

## Abstract

This paper presents the preparation and execution of on-ground static and engine load tests for the composite unmanned aerial vehicle (UAV). The test was conducted for pre-flight structural strength verification of the remotely piloted aerial target named HORNET, after introducing some structural modifications. The ground tests were performed before the flight test campaign, to ensure the strength and operational safety of the modified structure. The panel method and Computer Aided Design (CAD) modelling were adopted for numerical evaluation of aerodynamic and inertial forces’ distribution to simulate loading scenarios for launch, flight and parachute deploying conditions during the static test. Then, the multi-stage airframe static test was prepared and executed with the use of a designed modular test rig, artificial masses, as well as a wireless strain measurement system to perform structure verification. The UAV was investigated with 150% of the typical load spectrum. Furthermore, an engine test was also conducted on a ground test stand to verify strain and vibration levels in correspondence to engine speed, as well as the reliability of data link and the lack of its interferences with wireless control and telemetry. In the article, data achieved from the numerical and experimental parts of the test are discussed, as well as post-test remarks are given.

## 1. Introduction

The aircraft, manned or unmanned, is a complex technical object, which needs to maintain integrity in the wide-load spectrum. For that reason, before putting any flying object in the air, its airworthiness capabilities must be proven, both to prevent critical damage of the aircraft as well as potential material losses on the ground. Despite rapid advances in material engineering and mechanic strength analysis methods in modern software, the importance of experiments remains crucial for critical safety applications. This is true for common materials as well as for new materials and technologies, e.g., glass and carbon composites. Structural analysis and testing of a composite wing for an ultralight unmanned aerial vehicle (UAV) was considered by Sullivan et al. [1]. Structural integrity of some composite parts of a high-altitude long-endurance (HALE) class UAV was also evaluated by Frulla et al. [2]. The necessity of performing aircraft fatigue and static tests for structure strength design and evaluation was investigated by Jian Wu et al. [3]. Load distribution measurements are also conducted in other branches of industry, e.g., Lei Gao et al. [4] for large diameter pile. The overall reliability and maintenance analysis of UAV is delivered in Reference [5].

The first experimental step to verify structural strength is static loading tests, performed on the ground. This paper presents the preparation and execution of that type of pre-flight verification experiment on an example of a UAV used in the Polish Air Force [6] as remotely piloted aerial system (RPAS). Although that UAV has already been used for many years, proposed structural modifications and their influence on the fuselage integrity need to be verified before flight. The main modifications were: different winglet construction and their attachment to the wing, as well as front fuselage part prolongation to improve balance of the UAV. After positive results of the static test, the UAV will be prepared for in-flight campaign with a sufficient real time load monitoring system (example in Reference [7] for manned fighter-bomber). The operation of monitoring system elements will also be tested during static investigation.

To ensure high correspondence between loading for the static test and the operational spectrum, numerical modelling was utilized. Nowadays, the wide choice of numerical methods for modelling of aircraft aerodynamics allows to increase the model’s fidelity at the expense of computational time, starting with very basic methods, such as classic potential theory, up to Direct Numerical Solution (DNS) of the turbulent flow. The choice of method results is always a trade-off between accuracy of results and computational cost. The current problem of establishing pressure distribution on surfaces of the aircraft in various flight conditions requires (in some regions, fine-) grid discretization. On the other hand, exact values of aerodynamic coefficients, such as drag, are not required.

## 2. Materials and Methods for Test Preparation

### 2.1. Remotely Piloted Aerial System (RPAS) Platform Description

**Figure 1:**
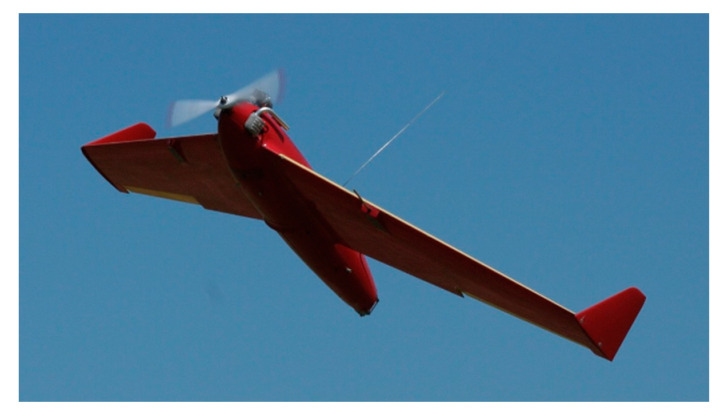
HORNET remotely piloted aerial system (RPAS) in-flight.

The RPAS named HORNET (Figure 1, Table 1) is part of the Aerial Target System, developed and deployed for operation in the Polish Army in 2005. Flying air targets are intended for training and performing artillery and artillery-rocket shooting by anti-aircraft defense forces.

The Aerial Target System consists of:

- Controlled maneuvering reusable air targets HORNET, equipped with a towed sleeve with an acoustic miss distance indicator and a towing line development system.
- Flight control and control systems allowing for remote control of “visual visibility” and command control, with the use of an on-board automatic control system.
- Starting launcher driven by rubber bands, with energy about 8 kJ.
- Transport system and a set of operating equipment.

From the start of the project, 47 pieces of the aircraft were produced. At present, due to airframe modifications to enhance performance and changes in the manufacturing process to increase product repeatability and precision, HORNET’s structure needs to be verified during ground and flight tests.

The flying wing aerodynamic configuration ensures high operational resistance in difficult conditions, as well as good performance at higher speeds and low mass of the structure. The maximum take-off weight is approximately 38 kg, including 5 kg of payload. To ensure the required performance, a 116 cm^3^, 11 house power, two-stroke two-cylinder spark-ignition piston engine was chosen. The take-off is carried out from the catapult (launcher) which ensures acceleration of the airframe with maximum take-off mass to the speed of about 74 km/h, with the maximum acceleration of about 150 m/s^2^.

**Figure 2:**
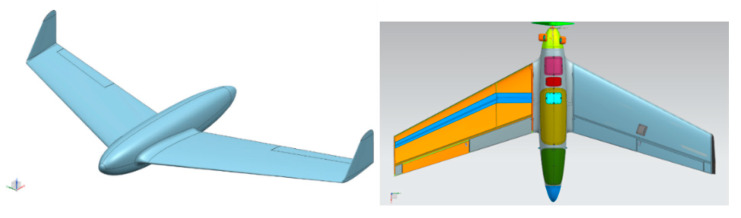
Geometry of “HORNET” unmanned aerial vehicle (UAV): skin (**left**) and internal structure (**right**).

The airframe of the aerial target is made using composite technology with sandwich structures formed in negative molds using vacuum techniques. The main material of the reinforcement is glass fiber, carbon cloth is used only for elements requiring stiffness and lightness (e.g., hatch cover, wing spar belts). Fuselage is circular and accommodates the engine, and fuel tanks are placed in the front as well as the battery for on-board systems. The middle part of the fuselage is a compartment for task equipment with servo-driven covers and a rolled-up shooting sleeve with the hit sensor. In the rear part of the fuselage, there is a parachute compartment with a servo-controlled cover. For transportation purposes, the plane is divided into the fuselage, wings and vertical stabilizers. The 140 cm long wings are connected to the fuselage using a circular connector made of duralumin. That duralumin connector in carbon mounting is an inner part of a front spar, until the second of 3 ribs. The back spar closes the wing profile with a working skin (Figure 2). There are also two fittings, placed on the leading and rear section of the socket ribs to transmit moments of forces.

### 2.2. UAV Numerical Model Preparation

The first part of the work was dedicated to preparation of the model, for reliable determination of aerodynamic forces on the ‘HORNET’ UAV. The data is required for evaluation of loads acting on the structure, so that a static experiment can be designed. Obtaining a good model requires performing a sequence of steps regarding geometry transformation into computational mesh. The process of model creation includes choice of physics, software, geometry and mesh generation, and finally, verification of the chosen method. The choice of potential flow method, also known as the panel method, was dictated by current needs. Often used for aircraft conceptual design process [8], the panel method’s advantages include high accuracy–computational cost ratio, ability to model required wing geometry and good prediction of pressure coefficient on wing surface.

On the other hand, there are some limitations of potential flow for modelling of aerodynamics. They are a result of assumptions, which panel codes make concerning flow physics [9], which are:

- Flow is steady,
- Viscosity is zero,
- Flow is irrotational,
- Flow is incompressible.

A number of panel method implementations are available for free, e.g., XFLR5, Panukl, etc., which are based on two-dimensional (2D) potential flow code XFoil [10,11,12].

“HORNET” is an UAV in a single wing configuration. The fuselage has conventional, streamlined geometry and is smoothly blended with the wing. The wing is tapered, swept, twisted and ends with a winglet. The NX CAD geometry is presented in Figure 2.

The bases for operational loading spectrum determination to conduct the static test were aerodynamic and inertial forces’ distributions from the CAD geometrical model (Figure 2). Additional information was achieved from collection of an on-board data recorder to specify the setting of the control planes for individual calculation variants.

Panukl allows to split the whole UAV geometry into smaller, easy to model parts, which can be merged afterwards. Fuselage is defined using point sets defining consecutive cross-sections. These can be directly imported from NX. The fuselage discretization is directly influenced by number of cross-sections, and points in a cross-section. Hence, the areas of higher curvature require finer spacing. The part of the geometry, which was simplified in this step, is the fuselage–wing blend. The main wing of “HORNET” was designed using NACA (National Advisory Committee for Aeronautics) profiles: root chord as NACA1410 and tip chord as NACA0008 with linear transition. The exact geometry was imported directly from NX via coordinates of points of key features:

- Wing sections at aileron start and end,
- Non-linear twist, resulting from constant leading-edge z-coordinate and linearly increasing trailing edge z-coordinate,
- Fuselage cross-sections.

The last part, namely wingtip, is modeled similarly to the main wing: NACA0006 profile is constant, and winglet is mounted at 90°.

Control surfaces are modeled by modification of airfoil, by rotating the trailing edge region around the hinge axis. This is a typical way of modelling control surfaces and was shown to give reliable results [13].

**Figure 3:**
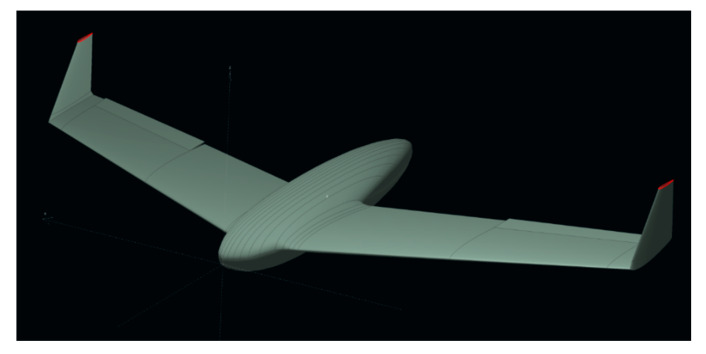
Isometric view of HORNET and fuselage with cross-sections defining its geometry.

**Figure 4:**
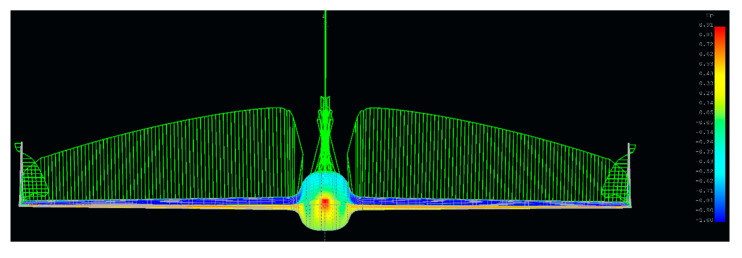
Lifting force distribution of the HORNET UAV.

Based on the above data, a family of models was created that responded to different configurations of aircraft aileron. Due to program limitations in the aspect of fuselage aerodynamic characteristics, it was modeled as a wing element, which can be seen in Figure 3. Then, the grid was developed and calculations of pressure and lift force distributions were calculated in correspondence to Figure 4.

Inertia loads were determined on the basis of mass distribution along the wingspan. The basis for these activities was a detailed HORNET model prepared in the NX software, illustrated in Figure 2. Using geometry from the CAD model as well as material data of individual elements, the distribution of masses was determined by dividing the wing in accordance with the geometry of the computational grid for aerodynamics. All concentrated masses and equipment built in the wing were also taken into account. Due to the distribution of the torsional moment of the wing, not only the distribution of masses along the wingspan but also the location of the centers of gravity of individual masses in the X-direction was taken into consideration in the numerical model.

**Figure 5:**
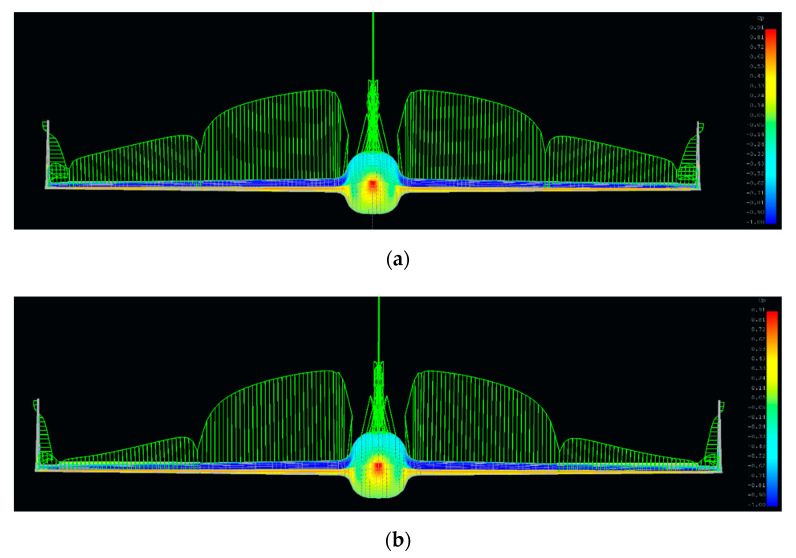
Aileron deflection influence on the aerodynamic loading distribution of the wing for (**a**) −5°, and (**b**) −10°.

Having all the necessary models, static test conditions were determined. Based on archival data from flights of the pre-modified version of the HORNET, the configuration of aileron during flights, including overload, was established. As a result of these analyses, it was determined that the most common values of the aileron is −5° for steady flights, while for stable flight fragments with Nz (vertical acceleration) above 5, the swing oscillates slightly around −10°. The influence of the aileron deflection on the aerodynamic loading distribution of the wing is presented in Figure 5. It can be noticed that for a higher aileron angle, the lifting force decreased for the outer part of the wing and the pressure point moved forward along the chord of the wing.

Another aspect taken into account was the effect of geometric dislocation and angle of attack on the load distribution. To obtain the maximum load states, in particular for bending moment, the maximum angle of attack from the aerodynamic model of 10° was taken for calculations. This allowed to achieve maximum loads on the outer parts of the wing and hence, the bending moment. Adoption of smaller angles would be associated with an almost complete loss of lift in the area of ailerons due to their upward deflection and negative geometrical dislocation of the wing, with a value of 3°. On the other hand, none of the above factors affected inertial loads.

**Figure 6:**
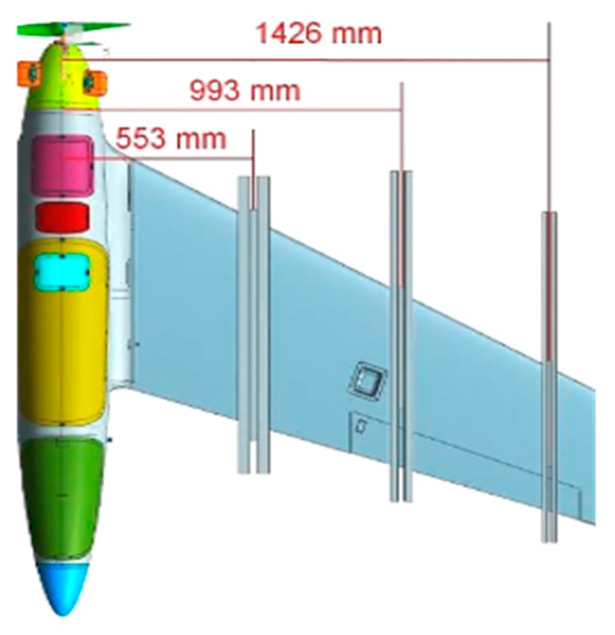
Position of the wing clamps.

After determining conditions to be met by the test, aerodynamic and mass loads were extracted from the models and the distribution of forces and moments derived from the assumed loads was specified. Afterwards, they were digitized to cross-sections in which the location of clamps was anticipated. The position of the clamps was selected on the distribution of the wing’s strength elements based on the CAD model (Figure 6). The use of a larger number of clamps was considered unnecessary due to the impossibility of mapping small loads on the external parts of the wing and limiting the transmission of concentrated forces by the wing itself.

**Figure 7:**
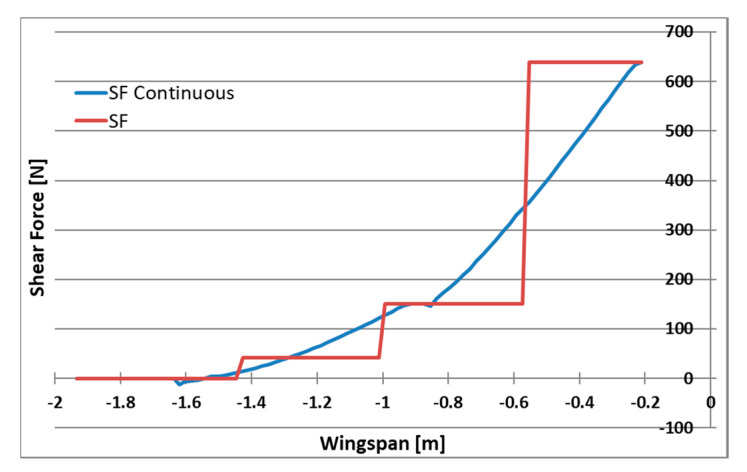
Shear force distribution for continuous and applied loading.

**Figure 8:**
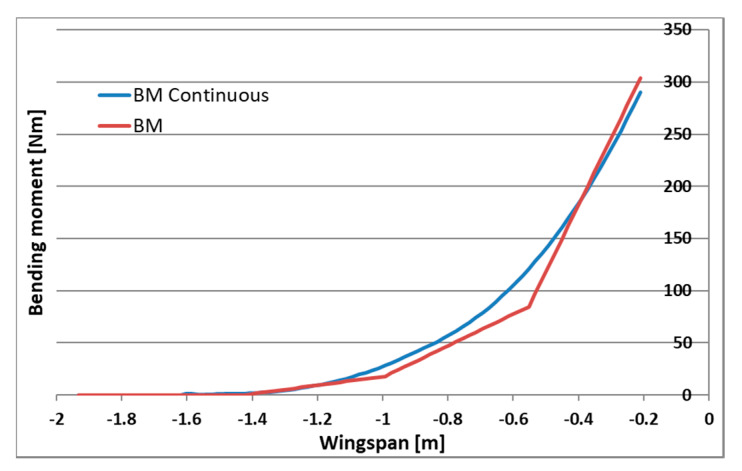
Bending moment distribution for continuous and applied loading.

**Figure 9:**
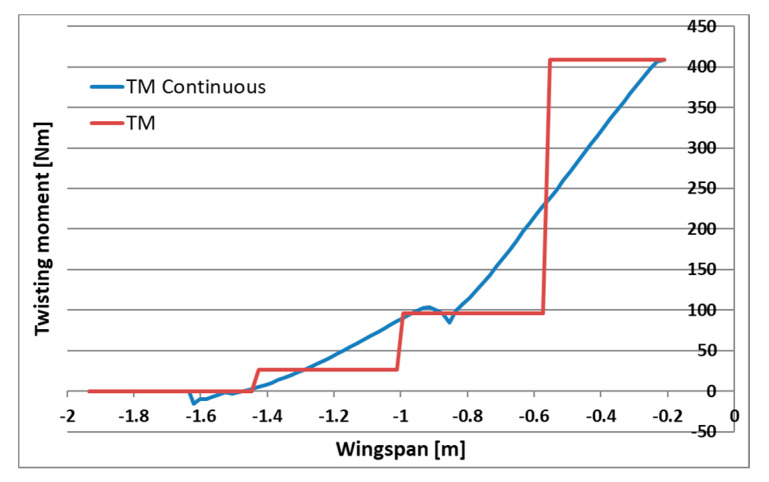
Twisting moment distribution for continuous and applied loading.

Numerical analysis of aerodynamic and inertial forces as well as geometry from the CAD model allowed for calculating their continuous distribution for the wing. A set of substitute masses has been selected for each UAV acceleration condition, which reflect the distribution of continuous forces with sufficient accuracy. The distributions were optimized for the largest possible matching of the bending moment distribution and not exceeding the loads at the base of the wing due to the load capacity of the fasteners. The comparison of continuous and applied loading is presented for shear forces, bending and twisting moments respectively, in Figure 7, Figure 8 and Figure 9. Load distribution for shear forces was calculated for the front spar, and the bending moment is transmitted by both front and back spar as well as by the working skin. The wing caisson experienced the twisting moment.

### 2.3. Preliminary Selection of Critical Areas of the Structure

**Figure 10:**
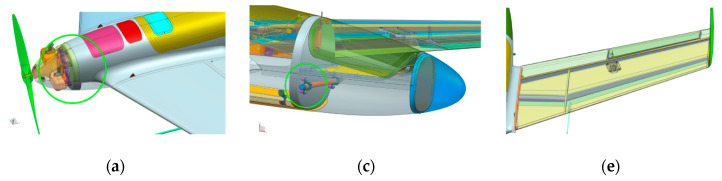

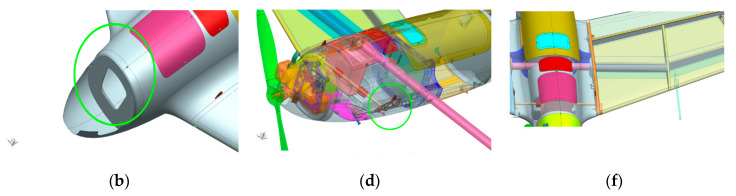
Critical areas of the Hornet UAV structure (**a**) propeller mounting area (**b**) propeller mounting frame (**c**) rear parachute mounting points (**d**) front parachute mounting points (**e**) wing with stabilizer (winglet) (**f**) wing structural elements.

The structure must be able to carry loads during landing at the permissible wind speed (hence the underdog system is not susceptible to damage). The most frequently occurring and significant structural damage of the HORNET aircraft is due to landing impact loads, including the parachute deployment phase. Due to unpredictable landing position and lack of landing gear, various areas are subject to the adverse effects of the landing impact, depending on the aircraft configuration during landing. The following areas are the most critical with respect to landing impact:

1. The propeller mounting frame (Figure 10a,b).
2. Parachute mounting points’ elements (Figure 10c,d).
3. Stabilizers (winglets) and wind-to-stabilizer mounting points (Figure 10e).
4. Wing structural elements—wing spar, especially wing-to-fuselage mounting points (Figure 10e,f).

### 2.4. Static Load Test Preparation

**Figure 11:**
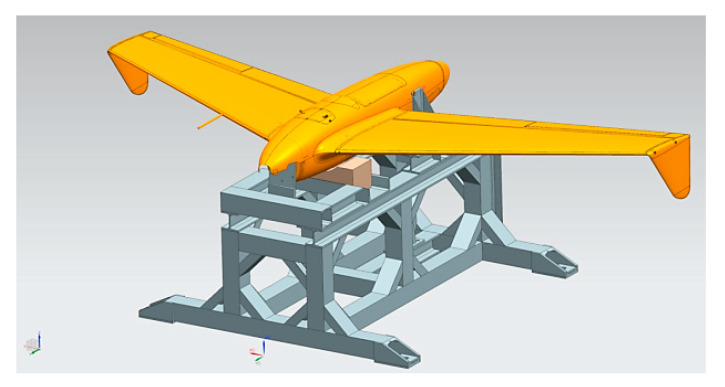
Test bed for static loading ground test configured for aerodynamical loads representation.

In order to ensure safety during flight tests on modified RPAS, pre-flight ground static tests were prepared and performed. These tests can successfully reveal structural design issues which can arise during flight tests and lead to potential failure and damage. The first critical part of the ground test is to apply static loads to the modified HORNET structure in order to investigate its capability to withstand loads expected to occur during flight. The load spectrum envelope was evaluated on the basis of historical flights of the standard version of HORNET as well as numerical research. The modular test rig was designed to enable investigation in various flight configurations (Figure 11). In addition, the static loads test of the HORNET structure was also used for the following tasks:

- Validation of the finite element model of the HORNET aircraft,
- Calibration and verification of components of the load monitoring system and acquisition of data useful for sensor network design during flight tests [14,15].

Airframe loading magnitude and the spread of strain and stresses during the static test correspond to those gained in the real flight. Apart from aerodynamic forces, another source of stress are vibrations due to a working engine. An additional quasi-dynamic test was also conducted to verify airframe response to vibration as a separate activity, which is also described further in this paper.

For the static on-ground testing, the investigation object was equipped with a sensor network based on foil strain gauges mounted to the platform skin for local measurements of the structure strain field. The suitable bridge configuration was chosen for the type of loads acting at a particular location and structural element [16].

**Figure 12:**
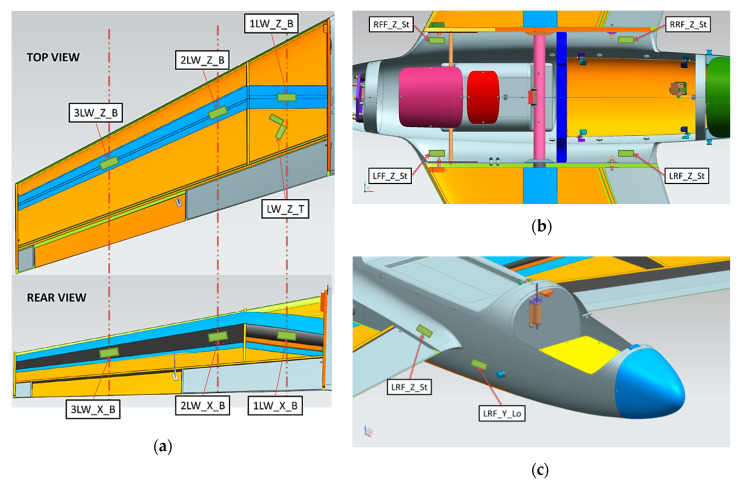
Strain gauges location (**a**) on the wing, (**b**) on the top fuselage and (**c**) on the side fuselage.

In Figure 12a, the location of the strain gauges on the HORNET wing is presented with top and rear views. Three measuring sections are marked, in which bending of the wing will be measured along the main spar. Bending is the structure’s reaction to lifting force during flight and maneuvers with vertical acceleration. The other three measuring sections are located directly on the main spar from the inner side of the wing, before the element will be fully manufactured. Those channels will allow for measuring front-to-rear bending of the wing, which occurs during take-off as a reaction to drag of the aircraft elements. Also, the change in torque of the wing is measured when the loading is set to several points along the chord of the airfoil section.

In Figure 12b, the location of the strain gauges on the top of the fuselage are presented. These front and rear measuring points will give the multi-type strain information during take-off and parachute deployment simulation. Additionally, the parachute fixing point is located near the front wing–fuselage connection points, which can produce significant strain on the fuselage during maneuvers. Sensors bonded behind rear wing-fuselage connection points should produce significant strain on the fuselage during maneuvers and take-off from the launcher. In Figure 12c, the location of the strain gauges on the side of the fuselage is presented. The measuring points will give the axial-longitudinal strain information during take-off and parachute deployment simulations.

In total, 20 analog input channels for strain gauges’ measurements were used, connected to wireless measuring modules by LORD Sensing MicroStrain to avoid routing long cables and ensure safety for test staff [17]. The sensors network was configured to investigate the magnitude and correctness of the loads at selected critical hot-spots during take-off, landing and parachute release. Additionally, the magnitude and correctness of the loads’ spacing along the airplane wingspan was also measured, using the optical three-dimensional (3D) scanning for deflection measurement of structural elements.

Several loading scenarios were planned for that test regarding load distribution and weight value to acquire discrete multipoint strain/load characteristics. In particular, the following type of loads will be represented on the HORNET structure.

### 2.5. Static Load Test Execution

The static loads test was divided into three stages:

- Stage 1—launch loads of the structure during take-off,
- Stage 2—flight loads of the structure during flight,
- Stage 3—parachute loads.

**Figure 13:**
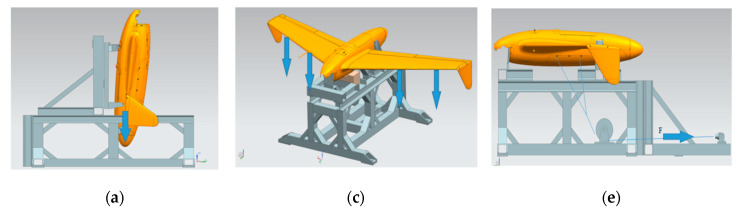

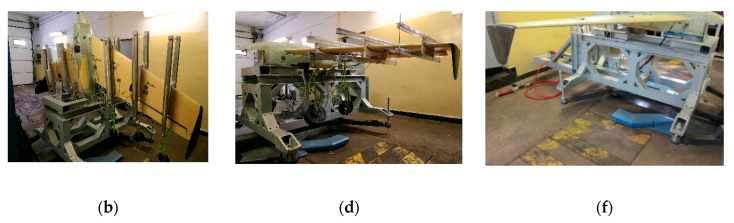
Test rig and RPAS configuration for all stages of static test (**a**) take-off (model) (**b**) take-off (execution) (**c**) flight (model) (**d**) flight (execution) (**e**) parachute release (model) (**f**) parachute release (execution).

The static loads were exerted to the airframe mounted in a modular test rig. Therefore, it allowed for installing the object in different positions according to the test stage (Figure 13). Since large loads are imposed on the structure during the launch by the four connection pins, these pins were used to mount the test specimen to the rig. No other support points on the fuselage were taken into consideration (besides a wooden block under the middle section of the fuselage, with the role of preventing the fuselage from critically deforming under higher loads). The total loads exerted on the aircraft structure are a superposition of aerodynamic loads and inertial loads due to maneuvers. The HORNET UAV structure is designed to be operated within <0; 6> vertical g-level. During the ground test, investigation was conducted to 150% of the maximum flight load, which corresponds to vertical load of order 9. The aerodynamic forces corresponding to the chosen flight conditions (in particular, corresponding with high vertical overload maneuvers) were computed using fluid dynamics. The main loading components taken into consideration to achieve real load patterns were: wing bending moment and shear force (which were controlled by adjusting weights attached to the clamps) as well as wing torque (which was controlled by location of the weight mounting point along the clamp). The following figures show the test rig in different configurations, corresponding to launch loads (Figure 13a,b) flight loads (Figure 13c,d) and parachute release loads (Figure 13e,f).

**Figure 14:**
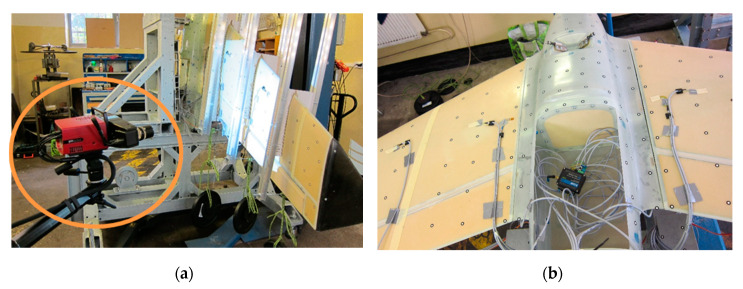
Structural deformation measurement with optical three-dimensional (3D) scanning system (**a**) camera (**b**) structure with reference point.

Together with strain measurements, the test object was covered with an array of markers (special stickers with contrast pattern) which allowed for optical displacement measurements of the structure under loads. The deformations were measured using an ATOS III digital scanner and are presented as displacements of the markers (Figure 14).

### 2.6. Engine Test Execution

**Figure 15:**
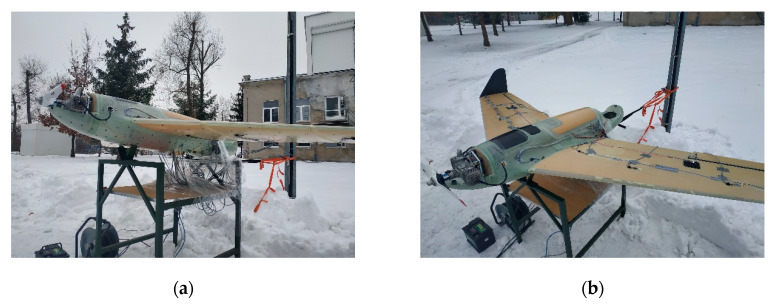
Hornet on the test stand for the engine test (**a**) bottom view, and (**b**) top view.

**Figure 16:**
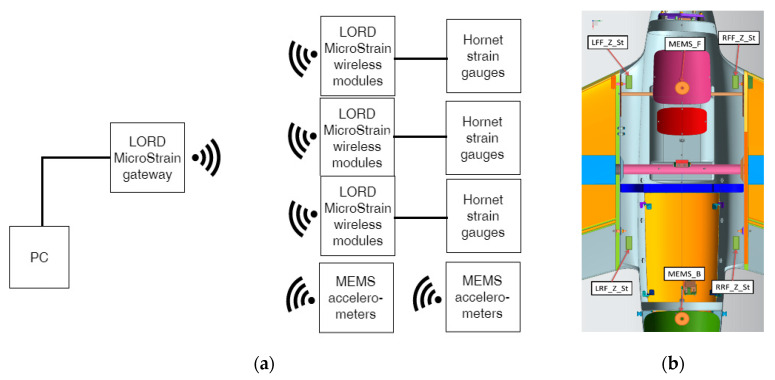
Engine test, (**a**) block diagram of wireless strain and acceleration measurement system, and (**b**) location of MEMS accelerometers on the fuselage.

After static test execution, an additional test was conducted with an UAV equipped with engine, avionics and electronics for control and telemetry. The engine test allowed for simultaneous determination of loading and vibrations from the engine as well as electromagnetic compatibility and lack of interferences between wireless communication of RPAS control and measuring equipment for in-flight load monitoring. This test will also allow to establish signal-to-noise ratio in the worst case scenario, when the aircraft is standing on the ground (Figure 15). Measurements were conducted for various engine speeds in the range of 1800–6400 revolutions per minute (RPM). The influence of vibration on equipment and its fixing was qualitatively evaluated. In the further stage of investigation during the flight test, this will lead to separate vibrations being derived from the engine and those from another source, like flatter. To do so, strain gauges bonded for the static test and additional MEMS (microelectromechanical system) accelerometers with a high sampling rate were used. For the sake of comparative character of research and type of used sensors, they were identical with anticipated flight configurations. In total, there were two 3-axis MEMS accelerometers as wireless modules, and 12 strain gauge channels grouped in 3 analog wireless modules (Figure 16).

An additional benefit from that test was a quality check of compatibility and a lack of electromagnetic interferences from RPAS control, telemetry and navigation with wireless measurement system. Both are wireless and use the frequency of 2.4 GHz. If both systems co-operate fine on their maximum emitted power with a presence of vibration on the ground, there should not be any interferences in the air.

In order to conduct the strain measurements for the aircraft structure both during on-ground and in-flight tests, wireless measuring modules by LORD MicroStrain V-Link and V-Link-200 were used. They are equipped with analog inputs and excitation voltage output sets for measuring bridges in which strain sensors are included. MEMS-type accelerometers are suitable for both ground and flight tests, because of their small size and low power requirement with integrated signal conditioning and temperature compensation. LORD MicroStrain G Link 200 contains 3-axis accelerometers of a range of up to 40 G and a 20-bit analog-digital converter acquisition system with a set of options (digital high- and low-pass filters) were utilized.

The presented measuring modules, independently from type, can be wirelessly connected to the data aggregator/base station. That device ensures wireless data acquisition from measurement modules and can be communicated through Ethernet or USB with PC via specialized software, Autonomous operation of the device and recording of the data on the internal memory of 16 GB is also possible.

## 3. Test Results and Discussion

### 3.1. Static Load Test

**Figure 17:**
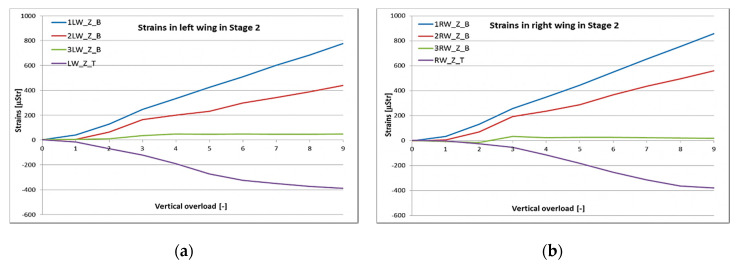
Strain measured in the (**a**) left and (**b**) right wing (bending in the *Z*-axis and torque) during Stage 2.

**Figure 18:**
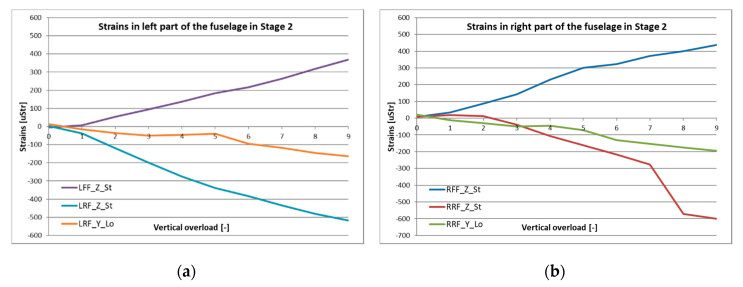
Strain measured in the (**a**) left and (**b**) right side of the fuselage during Stage 2.

During the test, weights corresponding to a particular load condition were hung on the clamps installed on the wings symmetrically on each side. For the flight load stage of the test, the aircraft was placed backwards in the horizontal position on the rig (Figure 13c,d). That configuration of the object allowed for use of only inertial forces acting on the structure, in order to achieve the lower vertical load conditions with no additional masses or clamps. The detailed load conditions used in this stage of the test are listed in Table 2. Results of strain measurements carried out in this stage for modified Hornet structure are presented in Figure 17 and Figure 18.

It can be noticed that changes in strain during this stage are considerable due to applied load. The linear increase of strain was measured with added mass in all cross-sections of the wing. Peak–peak value of strain from bending for inner and middle locations was around 800 and 500 microStrains (uStr), respectively (Figure 17). The decrease in peak–peak strain level corresponds with the modelling, where the strain levels are lower for the middle cross-section in correspondence with the inner one. The median difference during all loading conditions was around 0.43 for this stage of the test. For the outer section, strain change was much lower and remained almost constant after reaching nz = 3.

For the front and rear fuselage measurement points, similar positive (for tension) and negative (for compression) points can be seen. Strain value increase is linear with the loading condition, reaching around 400 uStr for front, and more than −500 uStr for the rear wing-fuselage fixing point (Figure 18). The exception with the rear right point could be explained by temporary keying of the structure in the test rig or a structure delamination (not confirmed by visual inspection).

**Figure 19:**
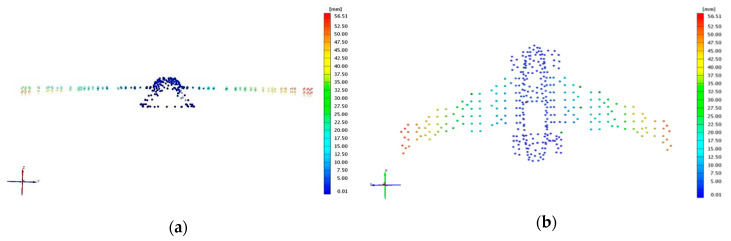
Comparison of structure displacements for nz = 1 and nz = 5 in the (**a**) horizontal and (**b**) vertical plane.

After installation of test specimens in the test rig and after applying desired loads to the structures, displacement measurements were carried out with the ATOS III scanner. A series of digital scans were performed allowing to determine the actual location of each marker. Using the baseline as well as location of markers placed on the rigid test rig (for solid reference), structural deflection was defined under a certain loading condition. The comparison for states nz = 1 and nz = 5 are illustrated in Figure 19. Especially in the horizontal view, the scale of wing deflection can be observed for those two loading conditions, reaching more than 50 mm for the outer end of the wing. There is almost no deflection of fuselage during that stage of the test.

**Figure 20:**
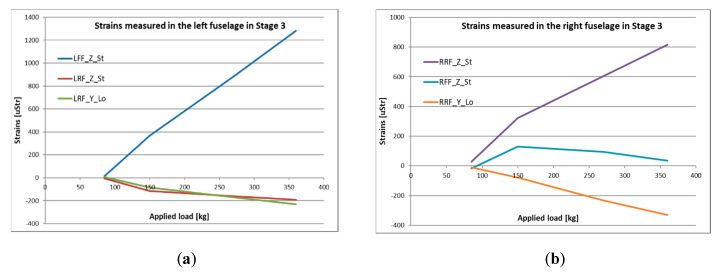
Strain measured in the (**a**) left and (**b**) right side of the fuselage during Stage 3.

Stage 3 was designed to verify the ability of the structure to withstand loads from deployment of the onboard parachute. Parachute mounting points in the front of the fuselage were used to attach lines to impose the loading. At this stage, the displacements were not measured with the optical system due to safety reasons. Figure 20 illustates strain data which were achieved for the modified HORNET structure until nz = 10. Despite the fact that the test was carried out to reach nz = 15 (Table 3), the hydraulic actuator was not able to stably maintain such high pressure for a prolonged time and strain measurements were not exact. However, the structure was able to withstand the test.

During the “parachute” stage of the test, the hydraulic actuator was pulling the parachute cords through a set of shackles and a force sensor. Several loading conditions were performed, applying the force in the range from 85 to 540 kg. The highest measured strain values were obtained for the front fixing points of the parachute lines with a magnitude of around 1000 uStr for nz = 10. Some differences in measurements between the left and right side of the fuselage can be noticed, but the character of those changes can be explained with a little asymmetry of the fuselage and test rig.

The main goal for the test was to evaluate structural strength for pre-flight validation. The structure withstood the applied loads with no critical damages to the structure. However, during visual inspection after the test, some damages were found, e.g., local delamination and cracks in the fuselage root rib area or cracks in the fuselage access. The damage was not critical, since the residual strength of the structure was high enough to carry the imposed loads (the failure was not critical). Those areas will be corrected for the in-flight campaign specimen to ensure structural integrity of the modified HORNET.

### 3.2. Engine Test

The engine test was performed on a test rig, with the mounting point similar to those from the UAV launcher. The test was conducted for various engine speeds in range of 2000–6400 RPM. For each engine speed, approximately 10 s of data was taken for further analysis, in which speed remained constant. Data from transient states have been removed to allow for comparison of time and frequency domain results for chosen engine speeds.

**Figure 21:**
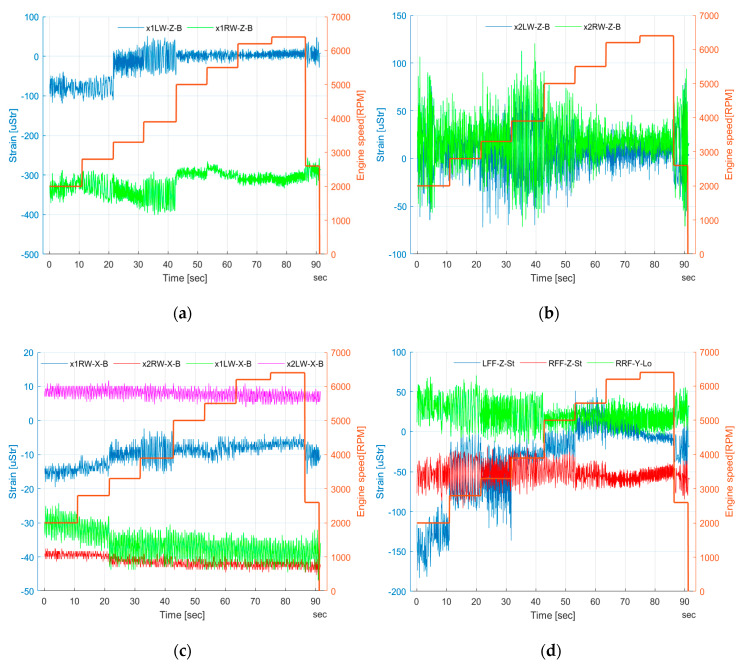
Strain measured on the wing and fuselage during the engine test (**a**) wing bending in the vertical plane—inner section (**b**) wing bending in the vertical plane—middle section (**c**) wing bending in the horizontal plane—inner and middle section, (**d**) fuselage parachute attachment points.

Strains measured on the wing and fuselage as well as corresponding engine speed are illustrated in Figure 21. For measurements from wing bending in the YZ plane (Figure 21a,b), some value alteration can be noticed. Strain alteration for the middle cross-sections 2LW_Z_B and 2RW_Z_B was about 150 uStr, with respect to RPM change. The highest peak–peak value can be seen for 3900 RPM for all channels. Regarding strain data from the inner cross-section, peak–peak range was as such for all RPM, but the median value increased with respect to RPM. Again, the highest range can be noticed for 3900 RPM.

Regarding measurements from wing bending in the horizontal plane (Figure 21c), the strain alteration with respect to RPM was almost negligible for all of the measurement channels. Peak–peak values were alike for all engine speeds. The slight change in mean level can be observed for the wing inner cross-section in the lower engine speed range. But, the overall change was approximately 10 uStr.

Alteration of strain gauges from the right parachute attachment point was relatively small, independent of engine speeds below 100 uStr peak–peak (Figure 21d). Strain gauge from the left front fixture showed a change of about 200 uStr.

The results from all measurement channels revealed some changes in both strain magnitude and its mean value, depending on RPM. The alteration was much higher for lower engine speeds, below 4000 RPM, even 2–3 times higher, in comparison to higher speeds, e.g., 6200 RPM. It can be explained by the high impact of the ground for the test object during that type of test as well as more “smooth” engine operation with higher RPM.

**Figure 22:**
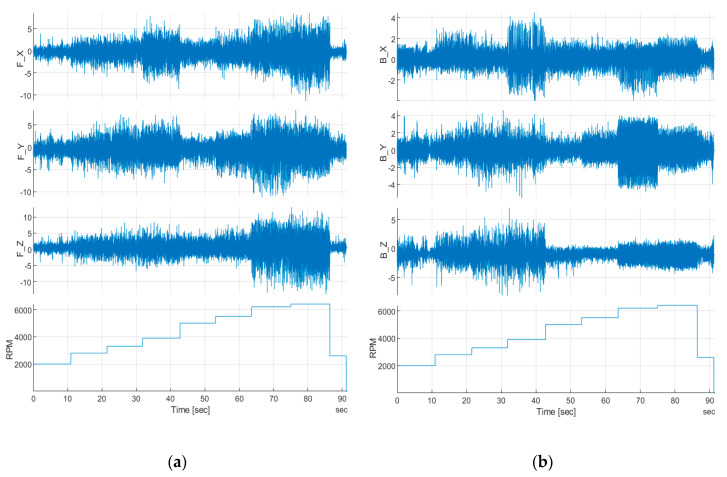
3-axis acceleration data versus engine speed for the (**a**) front, and (**b**) rear sensor.

Acceleration data illustrated in Figure 22 give information of vibration level in a function of engine speed from the front (Figure 22a) and rear (Figure 22b) 3-axis accelerometer. For RPM 2000–4000 peak–peak range for the rear of the fuselage, the acceleration is ±7.5 g for the *z*-axis, ±5 g for the *y*-axis and ±4 g for the *x*-axis. Above 4000, the envelope for each axis decreased by approximately 50%.

**Figure 23:**
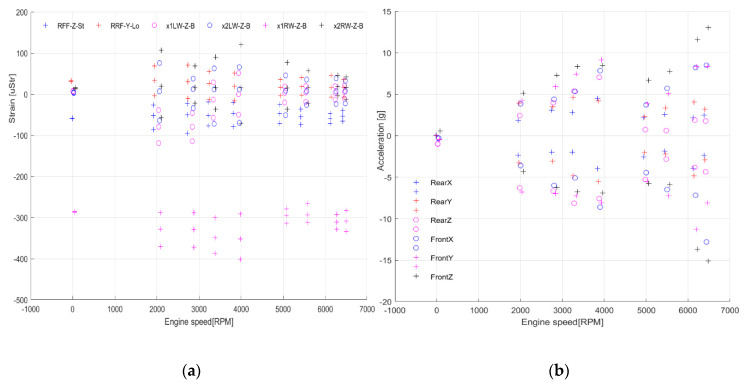
Max/median/min values during engine test for (**a**) strain sensors, and (**b**) accelerometers.

Regarding the MEMS accelerometer from the front of the fuselage (Figure 23a), an engine operation effect can be seen, especially for higher RPM. For 2000 and 2800 RPM, peak–peak value for all axes are quite similar, in contrast to the rear sensor, where the z, y and x axes sensed significantly different acceleration. The lowest envelope can be noticed for 5000 RPM and is fitted in the range of [+6; −5] for the *z*-axis and [+4; −5] for the y and x axes. Above 6000 RPM, a clear increase in the vibration envelope can be noticed for all sensing directions and was the highest for the test. Respectively, for the *x*-axis, in a range of [+8; −12] g, the *y*-axis [+8; −8] g and [+13; −15] g for the *z*-axis (Figure 23b).

Although the front sensor was mounted much closer to the UAV center of gravity than the rear one, the acceleration level was much higher and almost constantly increasing with respect to engine RPM. Acceleration measurements from the rear sensor confirmed (together with strain data) that higher structural vibration occurs on the ground for engine speeds lower than 4000 RPM. Also, the negative influence of engine operation can be decreased by mounting accelerometers closer to the rear end of the fuselage, so behind the UAV center of gravity. If not, a special dumping platform should be considered to reduce engine vibration on acceleration measurement.

**Figure 24:**
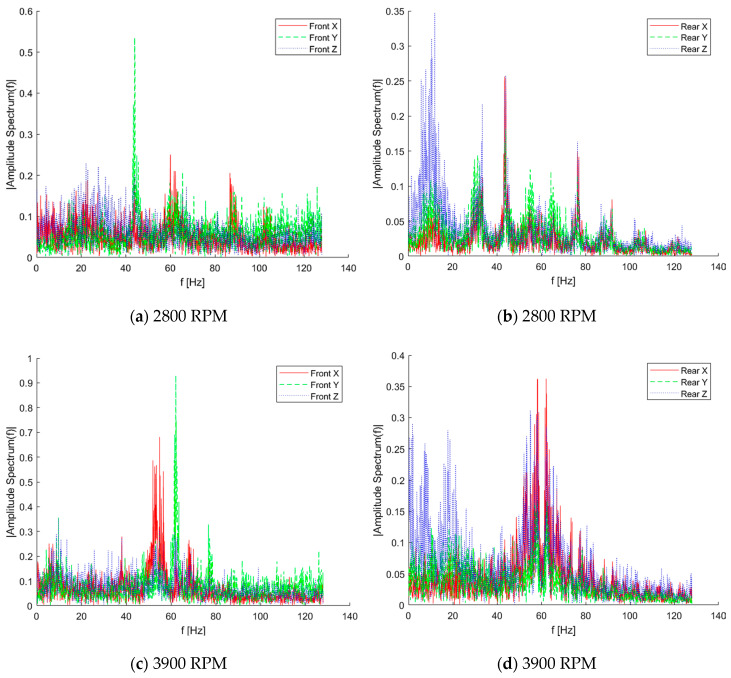

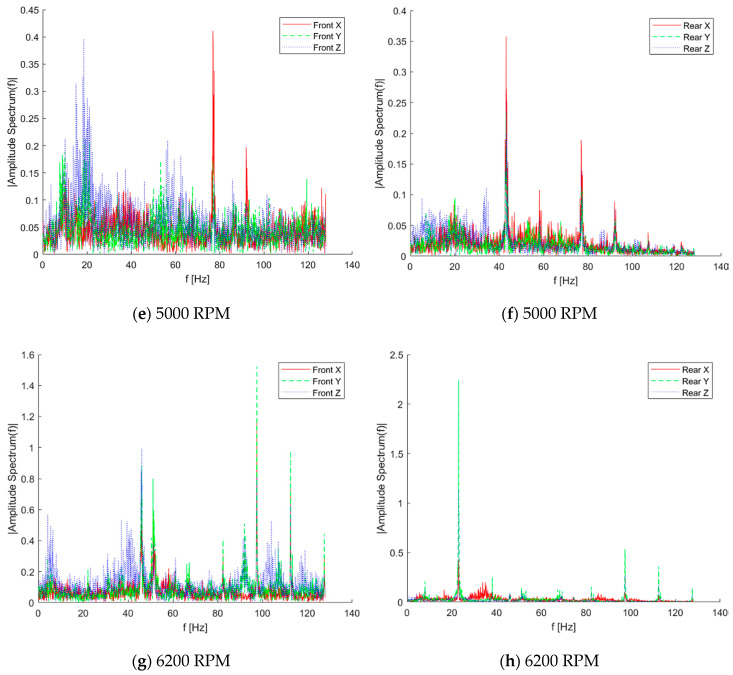
3-axes acceleration for selected engine speed for sensor (**a**) front—2800 RPM (**b**) rear—2800 RPM (**c**) front—3900 RPM (**d**) rear – 3900 RPM (**e**) front—5000 RPM (**f**) rear—5000 RPM (**g**) front—6200 RPM (**h**) rear—6200 RPM.

In Figure 24, amplitude spectrums based on Fast Fourier Transform (FFT) for both front and rear 3-axis acceleration sensors are illustrated for selected engine speeds. The spectrum decomposition and frequency analysis confirmed remarks from time domain signals. For the rear sensor, there were significant vibrations for 2800 and 3900 RPM (Figure 24b,d) within the range 1–25 Hz, mostly from the *z*-axis. Additionally, some resonance effects can be observed for the speed of 3900 RPM, as a delta-type envelope around the dominant, rotational frequency for all 3 axes. Above that speed (5000 RPM and 6200 RPM), the spectrum become more flat, and low-frequency components were strongly decreased, similarly for all axes. Sharp peaks on the charts correspond with dominant, rotational frequency and its subparts (Figure 24f,h).

Regarding the front accelerometer, some frequency components in a range below the dominant one for engine speed increase in the amplitude spectrum, in line with rotational frequency. For lower engine speeds (2800 and 3900 RPM), the envelope for all 3 axes was similar in that range. For the main dominant frequency, the *y*-axis component had the highest level. With the further increase of engine speed, the *z*-axis envelope became significantly more elevated in regions outside the rotational frequency than the *x*-axis and *y*-axis envelope. The character of frequency domain data affirms that the front acceleration sensor is strongly affected by engine operation. The level of vibration on frequencies other than rotational speed can have several sources: moving parts in the two-stroke, two-cylinder, spark-ignition piston engine itself, local resonance of the structure (frames, internal shelfs) due to external excitation, as well as multibody effects from a test stand–UAV connection. But, on the contrary to the rear sensor spectrum, those effects occur and are stronger with the increase of engine speeds.

Summing up, the engine test revealed that there is no significant change in strain of wing bending in the XY plane corresponding to engine speed. Bending in the YZ plane showed some alterations with respect to engine speed, which is higher for the inner cross-section. On the other hand, peak–peak range changed significantly for acceleration and the envelope was higher for lower RPM for rear fuselage. In each case, the *z*-axis showed the biggest alteration. For the front fuselage, the engine effect can be seen, as in the lower RPM region, the envelope was alike for all axes. The envelope for all sensing directions increased significantly with respect to engine speed. Furthermore, the operation reliability of the measurement system was confirmed in the presence of external disturbances from engine vibration.

The supplementary information from the engine test was the evaluation of the joint operation of two wireless data links: one for UAV control and telemetry and the second for a strain and acceleration monitoring system for future activities during the flight test. Real-time load monitoring system implementation for the aircraft is currently one of the main tasks to ensure the optimal maintenance and utilization strategy as a part of health and usage monitoring for technical objects [7,18] for the individual aircraft tracking (IAT) concept. Regarding wireless data links, one of the concerns was the fact that both are using the same frequency of 2.4 GHz. During the engine test, simultaneously with strain and acceleration data, the Received Signal Strength Indicator (RSSI) parameter was acquired, which is the measure of the power present in a received radio signal. The RSSI value is measured in dBm, in their response packet. For the chosen measurement system, this signed 1-byte value can range from −95 dBm to +5 dBm. The Node RSSI is the signal strength that the node received the command from the base station. The Base Station RSSI is the signal strength that the base station received the response back from the node. Both Node and Base RSSI values were within the range of −14 to −30 dBm and were unaffected by the operation of the engine. The test also proved no negative effect on RPAS on-board primary equipment due to wireless measurement system utilization. The short- (few meters) and long-range (100 m) test of control equipment was performed and no interferences within both wireless data links were experienced.

## 4. Conclusions

This paper presented an on-ground static load test as well as engine test preparation and execution for UAV pre-flight structural strength verification and wireless real time monitoring system reliability. The loading spectrum for the static test was evaluated using historical data of a non-modified version of the HORNET UAV and numerical modelling with the use of the panel method. The novel approach during that activity was that the fuselage was modeled as a part of the wing. As a consequence, better estimation of load distribution was achieved in the wing–fuselage connection area. Furthermore, the influence on aerodynamic load distribution along the wingspan due to different aileron angles was taken into consideration. This is also quite important, when investigating UAV in tailless configuration, where lift is generated on wings.

The ground test was performed with the use of a specially designed modular test bed, which could be quickly adapted to carry out test scenarios for different flight stages. The structure load spectrum was prepared to be higher than the expected envelope during flights up to the ultimate load for that airframe. Strain measurements from the wing bending confirmed load distribution, in which strain magnitude was significantly higher for the inner part of the wing during the static test, but low values of strain for the wing outer section were caused by the load application method. Under actual operational aerodynamic loads, these values should be enhanced, which must be taken into account while designing winglet fixing points. Additionally, utilization of a 3D optical scanner together with strain measurement gave the information of continuous deflection of the fuselage and wings during loading. The 3D scanner can also be useful to monitor pre- and post-test object geometry, which could disclose potential plastic deformation of the airframe. Based on the visual inspection, no critical damage was noticed after the test was completed. The airframe withstood the applied loads in all 3 test stages.

Based on the results of the performed tests, the following conclusions can be drawn:

- Structural damages found after completion of the tests were not critical, however would probably propagate during operation. These locations (Critical Points) should be monitored during regular operation.
- Strain gauges intended to measure strains resulting from front-to-rear wing bending showed relatively low values. This might be caused by wing design, in which loads are mainly transferred by the leading and trailing edge of the wing.
- The engine is the main source of vibration for the structure and can significantly influence sensors’ reading, especially for those located in front of the fuselage. Sufficient damping platforms should be applied to reduce that effect, in particular, for acceleration sensors.
- Simultaneous operation of two wireless data links, for control and telemetry and for the strain and acceleration monitoring system, was confirmed to be reliable and can be safely used during flight tests in the near future.

Based on the positive results of presented tests, the next stage of the research will cover the flight test campaign. That will be the final test for HORNET UAV structure as well as for the integrated load monitoring system, which can become a permanent part of the on-board equipment of that aircraft.

## Figures and Tables

**Table 1:** HORNET RPAS basic parameters (min = minimum, max = maximum).

Length/wing span	1.7 m/3.2 m
Starting weight/payload	38 kg/5 kg
Min/cruise/max speed	85/150/230 km/h
Operating range	40 km
Max climb speed	16 m/s (at 140 km/h)
Parachute drop velocity	5.5 m/s

**Table 2:** Load conditions used in Stage 2—flight loads.

Load Condition	Inner Clamps (kg)	Middle Clamps (kg)	Outer Clamps (kg)
	Total Weight	Total Weight	Total Weight
**nz = 1**	7.17	0.00	0.00
**nz = 2**	14.92	2.87	0.00
**nz = 3**	22.67	4.43	1.80
**nz = 4**	31.17	6.43	2.15
**nz = 5**	39.42	8.43	2.65
**nz = 6**	47.67	10.18	3.40
**nz = 7**	55.92	12.18	4.15
**nz = 8**	64.17	13.93	4.90
**nz = 9**	72.42	15.93	5.65

**Table 3:** Load conditions used in Stage 3—parachute release.

Load Condition	nz = 2.5	nz = 5.0	nz = 7.5	nz = 10.0	nz = 12.5	nz = 15.0
**F (kg)**	85	150	270	360	450	540
